# Association between levels of blood trace minerals and periodontitis among United States adults

**DOI:** 10.3389/fnut.2022.999836

**Published:** 2022-09-07

**Authors:** Handan Huang, Jingjing Yao, Nan Yang, Liuqing Yang, Lu Tao, Jinling Yu, Ying Gao, Zhihui Liu

**Affiliations:** Department of Prosthodontics, Hospital of Stomatology, Jilin University, Changchun, China

**Keywords:** trace minerals, periodontitis, selenium, manganese, lead, cadmium, mercury, NHANES

## Abstract

**Aim:**

Evidence linking trace minerals and periodontitis is limited. To investigate the relationship between trace minerals (selenium, manganese, lead, cadmium, and mercury) and periodontitis, data from the National Health and Nutrition Examination Survey (NHANES) were analyzed after accounting for potential confounding factors. No known studies have explored the relationship between these five trace minerals and periodontitis.

**Materials and methods:**

A total of 4,964 participants who had undergone a full-mouth periodontal examination and laboratory tests for five trace minerals were studied in a cross-sectional study. Clinical attachment loss (CAL) and periodontitis grading were used to measure periodontitis severity. Linear and logistic regression models were used to evaluate the association between trace minerals and periodontitis. Further subgroup analyses were performed.

**Results:**

Blood lead and cadmium levels were positively associated with mean CAL, and blood selenium was negatively associated with mean CAL; however, blood mercury, blood manganese, and mean CAL were not significantly associated. The association between trace minerals and mean CAL was more significant in males, the elderly, and patients with diabetes. There was a threshold effect between blood cadmium levels and mean CAL. Among the Black population, the relationship between blood cadmium levels and mean CAL followed an inverted U-shaped curve. There was a saturation effect in the study of blood lead in people aged 45–59 years old.

**Conclusion:**

Our study highlighted that blood selenium, lead, and cadmium levels were significantly associated with periodontitis in a nationally representative sample of United States adults.

## Introduction

Periodontitis is a common, microbially generated, chronic inflammatory illness defined by the loss of alveolar bone and supporting periodontal ligament. Approximately 10.8% (743 million) of people worldwide had severe periodontitis in 2010, making it the sixth most prevalent health condition (1). A study showed that between 2009 and 2012, 46% of American adults had periodontitis, and 8.9% had severe periodontitis (2). Periodontitis can cause periodontal abscesses and tooth loss. Several systemic diseases are known to be strongly associated with periodontitis. Patients with periodontitis are at higher risk of developing and/or exacerbating diabetes, Alzheimer’s disease, chronic obstructive pulmonary disease, and cardiovascular diseases (3, 4). The finding of comorbidity between periodontitis and various medical disorders has raised periodontitis to a new level of importance and has become a worldwide health issue.

Periodontitis is primarily driven by an exacerbated host immune-inflammatory response, which has many drivers (5). Trace mineral imbalances may contribute to the causal pathway in some patients. Many trace minerals are related to antioxidant properties and oxidative stress, which are associated with periodontitis. Selenium and manganese are essential minerals in the body and are found mainly in foods such as nuts and fish. Periodontal tissue benefits from selenium primarily because of its antioxidant action. Selenium and selenoproteins participate in immune regulation and prevent overreactions that can cause autoimmunity or chronic inflammation (6). In patients with diabetes and low selenium levels, chronic periodontitis progresses (7). Thus, selenium has a protective effect on periodontal tissues. Manganese participates in various biochemical functions, such as immune function, growth, and development, and serves as a component of reactive oxygen species (ROS) detoxification (8). Manganese is a cofactor for several enzymes, including oxidoreductase and glutamine synthetase, and protects organisms from oxidative stress (9).

Lead, cadmium, and mercury are considered potentially harmful trace elements but may have beneficial effects at low concentrations. Lead exposure can adversely affect bone metabolism and the immune system, which are potential risk factors for periodontitis. Long-term lead exposure may damage the periodontal ligaments, gums, and alveolar bones (10). Higher blood lead levels have been found to be significantly associated with a decline in adult periodontal health (11). Cadmium exposure has numerous effects (12). Cadmium increases bone resorption, inhibits osteoclast activity, reduces calcium absorption, and impairs kidney function, thereby promoting osteoporosis (13). Moreover, cadmium exposure induces the production of ROS, which may be a potential cause of periodontitis progression (14, 15). In a rodent model of long-term cadmium poisoning, cadmium has been shown to cause loss of the dental alveolar bone (16). Apart from food and fish, dental amalgam is a particular source of mercury, which was popular in the past; however, it is now being reduced and eliminated (17). Despite the low levels of Hg in the environment, its bioaccumulation, toxicity, and persistence may still threaten human health (18). Mercury exposure increases the production of ROS, free radicals, and superoxide anions (19). Wildemann et al. (20) reported that the critical role of mercury-induced toxicity is to inhibit the antioxidant defense system, change the oxidation-antioxidant balance, and increase ROS, which may aggravate damage to periodontal tissue.

To our knowledge, there have only been two early studies published, both ten years ago, that used NHANES III data (1988–1994), focused on the relationship between single trace minerals (lead and cadmium) and periodontitis among adults in the United States of America (21, 22). There is limited evidence linking trace minerals to periodontitis. Thus, this study investigated whether the blood levels of the five trace minerals were associated with periodontitis and whether the blood trace mineral level showed a dose-response relationship. To this end, we conducted a cross-sectional study using data from the United States NHANES dataset.

## Materials and methods

### Ethics statement

This study was exempt from Institutional Review Board review, for we used data that were deidentified from the NHANES database. Additionally, the protocols for NHANES 2011–2012 and NHANES 2013–2014 were approved by the National Center for Health Statistics Ethics Review Board, a part of the Centers for Disease Control and Prevention (CDC). Prior to the commencement of the survey, all participants were required to provide written informed consent. The NHANES assesses the health and nutritional status of adults and children in the United States, which is a cross-sectional, stratified, multi-stage study. In our analysis, we combined data from two NHANES cycles (2011–2012 and 2013–2014).

### Study population

**FIGURE 1:**
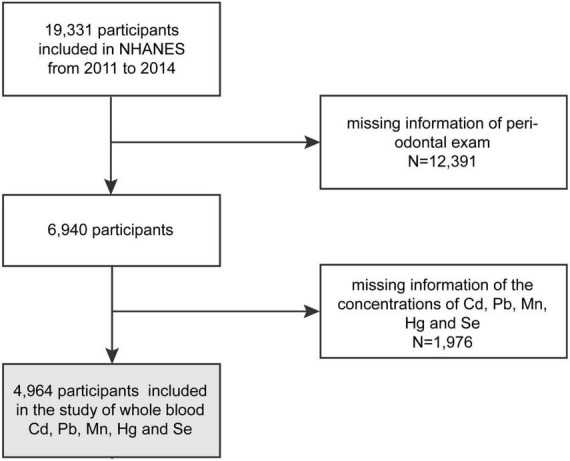
Flow diagram of selection of the study participants, from NHANE 2011–2012 and 2013–2014.

An analysis of NHANES 2011–2012 and 2013–2014 data was conducted to evaluate the health and nutritional status of a representative sample of non-institutionalized people in the United States. Survey participants were asked to complete household interviews and physical examinations in mobile examination centers (MEC). NHANES is a publicly available dataset, and more details of methods and other information are available at www.cdc.gov/nchs/nhanes/. In the NHANES cycles included in this study, 19,331 participants were interviewed. A total of 6,940 adults aged 30–80 with periodontal exams constituted the study sample. We further excluded those who had not yet completed blood laboratory tests for mercury, cadmium, selenium, manganese and lead, leaving 4,964 individuals in the analysis cohort (Figure 1). The missing values of continuous covariates (vitamin D and Ca intake) were less than 0.3%; these were replaced by the average value, and the missing values of classified covariates were stratified based on missing-data status.

### Periodontal examination

If a participant aged over 30 had at least 1 natural tooth, a full-mouth periodontal examination was conducted. All dental examiners were trained and calibrated by the survey reference examiner. The pocket depth and gingival recession at 6 sites per tooth were measured with a color-coded periodontal probe which graduated in 2-mm increments. Professional inspectors examined all four quadrants and the measurements were rounded to the full millimeter. In the data entry program, the clinical attachment loss (CAL; distance between the cementoenamel junction and the bottom of probing pocket) was automatically calculated by the algorithm. The result variables of this analysis were mean CAL and the severity of periodontitis. The grade according to the severity of periodontitis is shown in Table 1 based on Eke et al.’s research (23).

**TABLE 1:** Definitions of periodontal status according to the severity of periodontitis.

Periodontal status	Definition
Severe periodontitis	≥2 interproximal sites with AL ≥6 mm (not on same tooth) **and** ≥1 interproximal site with PD ≥5 mm
Moderate periodontitis	≥2 interproximal sites with AL ≥4 mm (not on same tooth), **or** ≥2 interproximal sites with PD ≥5 mm (not on same tooth)
Mild periodontitis	≥2 interproximal sites with AL ≥3 mm, **and** ≥2 interproximal sites with PD ≥4 mm (not on same tooth) or one site with PD ≥5 mm
Noperiodontitis	No evidence of mild, moderate, or severe periodontitis

### Blood sampling

During the laboratory exam, whole blood specimens were collected, shipped on dry ice to the National Center for Environmental Health, Atlanta, GA, and stored frozen at 20°C until analysis. Whole blood concentrations of essential (selenium and manganese) and potentially toxic elements (lead, cadmium and mercury) were measured using inductively coupled plasma mass spectrometry (ICP-MS) after a simple dilution sample preparation step. Detailed analysis methods can be found on the NHANES website. Following all quality control procedures recommended by the manufacturer, the results of all measurement reports met the Division of Laboratory Science’s quality control and quality assurance performance standards for accuracy and precision, similar to the Westgard rules. For all biomarkers, according to the NHANES protocol, the concentration below the detection limit was divided by square root 2.

### Other covariates

We also collected other covariates. The gender was male or female. The age of the participants was from birth to the date of interview. Race/ethnicity was classified as: non-Hispanic White, non-Hispanic Black, Mexican American, and other race/ethnicity. The educational level of the participants was classified as below high school, high school or above. The poverty-to-income ratio was calculated by dividing family income by the yearly poverty threshold published by the Census Bureau. The poverty ratio was categorized into three levels: ≤1; 1–3; >3. Smoking status was categorized as never smoking (lifetime smoking less than 100 cigarettes), current smoking (lifetime smoking more than 100 cigarettes and smoking during the survey period) and former smoking (lifetime smoking more than 100 cigarettes but not smoking during the survey period). Participants with diabetes were recorded if they met one of the following criteria: fasting blood glucose ≥7.0 mmol/L, 2-h blood glucose ≥11.1 mmol/L, glycated hemoglobin ≥6.5% and self-reported diabetes diagnosis. Body mass index (BMI) was the data obtained by dividing weight (kg) by the square of height (m). BMI was classified as underweight, normal weight, overweight, or obese based on BMI <18.5, 18.5–25, 25–30, or >30, respectively. In MEC, blood samples were collected through venipuncture and tested for serum 25(OH)D levels using the standard protocol. Bone mineral density (BMD) was measured at the lumbar vertebrae. The levels of BMD were categorized using quartiles (quartile 1: <25th percentile, quartile 2: 25th–50th percentile, quartile 3: 50th–75th percentile, quartile 4: >75th percentile). We based on the response to the questions in NHANES: In the past week, how many days have you/SP used dental floss or other devices to clean your teeth besides brushing your teeth with a toothbrush? (OHQ.870); How many days have you/SP used mouthwash or other mouthwash products to treat dental diseases or problems in the past week? (OHQ.875). Four categories were created from the responses: from 0 day, 1--2 days, 3--4 days, and 5 or more days in the last week. Periodontal treatment was divided into ‘‘yes’’ or ‘‘no’’ according to the participants’ response to the question, ‘‘Ever had treatment for gum disease?’’. The presence of hypertension and hyperlipidemia was assessed by the questions of the interview, ‘‘Ever told you had high blood pressure?’’, ‘‘Doctor told you high cholesterol level?’’. Dietary recall interviews were collected in person in the MEC. Daily calcium intake was then calculated. Based on the 2008 Physical Activity Guidelines for Americans,^1^ four levels of physical activity were created: (1) sufficiently active: engaging in ≥300 min/week of moderate activity, or ≥150 min/week of vigorous activity, or ≥300 min/week of an equivalent combination; (2) active: engaging in ≥150 min/week of moderate activity, or ≥75 min/week of vigorous activity, or ≥150 min/week of an equivalent combination; (3) insufficiently active: reporting some physical activity, but not enough to meet the active definition; (4) inactive: no physical activity. Responses to questions of the interviews were used to assess the presence of congestive heart failure, coronary heart disease, angina pectoris, heart attack, stroke, and physical activity. Detailed information regarding each particular covariate is available at www.cdc.gov/nchs/nhanes/. If a given covariate resulted in a change in effect estimate of more than 10% (24), or was significantly associated with mean CAL, the variable was chosen as a confounder. The above covariates were selected *a priori* based on established associations and/or reasonable biological relationships and tested.

### Statistical analyses

All analyses were conducted using sampling weights based on the recommendation to interpret the complex NHANES survey design. Continuous variables were expressed as the means ± standard deviations and compared using the t-test. Categorical variables were expressed as percentages and compared using the chi-square test. Linear regression models were used to evaluate the association between the concentrations of the five trace minerals and the mean CAL. Continuous variables of trace mineral concentrations were converted to quartile variables to perform trend tests for a robust analysis in the models. Subgroup analyses were also conducted. A generalized additive model and smooth curve fitting method were used to identify the non-linear relationship. When non-linearity was detected, the turning point was calculated using the recursive algorithm, and then a piecewise linear regression model was constructed to calculate the threshold effect and saturation effect of trace minerals on mean CAL. Multinomial logistic regression models were used to evaluate the association between trace mineral concentrations and periodontal status when data did not pass the test of parallel lines. As recommended by the STROBE statement, we examined the results of the unadjusted or minimum adjustment analysis in parallel with those of the fully adjusted analysis. Model 1, unadjusted; Model 2, age, sex, and race/ethnicity were adjusted; Model 3, age, sex, race/ethnicity, BMI, income-poverty ratio, education, vitamin D, smoking status, diabetes, frequency per week using floss and mouthwash, periodontal treatment, hypertension, hyperlipidemia, BMD, congestive heart failure, coronary heart disease, angina pectoris, heart attack, stroke, physical activity, and calcium intake were adjusted. All of these analyses were performed using the statistical software packages R^2^ (The R Foundation), SPSS (Version 22.0 for Windows; SPSS Inc.), and EmpowerStates^3^ (X & Y Solutions, Inc., Boston, MA, United States). *P* values less than 0.05 (two-sided) were considered statistically significant.

## Results

### Baseline characteristics of participants

A total of 4,964 subjects were enrolled in our study (Figure 1), of whom 2,509 met the criteria for periodontitis. Table 2 showed the weighted sociodemographic and medical characteristics of the participants with and without periodontitis. Of all participants, the mean age was 51.04 ± 13.52 years, 48.99% were males, 68.59% were White, 10.14% were Black and 8.04% were Mexican-American. Among the different groups with and without periodontitis, age, sex, race/ethnicity, education, income-poverty ratio, vitamin D, smoking status, diabetes, frequency per week using floss and mouthwash, periodontal treatment, hypertension, calcium intake, congestive heart failure, coronary heart disease, angina pectoris, heart attack, stroke, physical activity and BMD were all significantly different.

**TABLE 2:** Characteristics of the 4,964 participants with and without periodontitis.

Characteristics	All	No periodontitis (*n* = 2455)	Periodontitis (*n* = 2509)	*P-value*
Age (years)	51.04 ± 13.52	48.53 ± 12.84	54.57 ± 13.67	<0.01
Male (%)	48.99	42.19	58.54	<0.01
Race (%)				<0.01
Non-Hispanic White	68.59	74.71	59.97	
Non-Hispanic Black	10.14	7.56	13.76	
Mexican American	8.04	6.08	10.8	
Other race	13.23	11.64	15.47	
Education (%)				<0.01
<High school	14.4	8.75	22.35	
High school	20.83	16.46	26.96	
>High school	64.76	74.78	50.67	
Poverty-income ratio (%)				<0.01
<1	11.49	7.77	16.72	
1–3	32.1	26.12	40.5	
>3	50.51	60.86	35.96	
Not recorded	5.9	5.25	6.82	
BMI (%)				0.22
Underweight	0.34	0.27	0.43	
Normal	26.25	27.23	24.86	
Overweight	36.18	36.85	35.24	
Obese	36.75	35.27	38.83	
Not recorded	0.49	0.37	0.64	
BMD (%)				<0.01
Quartile1	15.47	16.36	14.23	
Quartile 2	15.36	15.68	14.91	
Quartile 3	15.74	18.26	12.21	
Quartile4	15.89	18.33	12.46	
Not recorded	37.53	31.37	46.19	
Vitamin D (nmol/L)	72.31 ± 29.02	75.01 ± 28.78	68.53 ± 28.93	<0.01
Smoking status (%)				<0.01
Never	55.58	63.54	44.4	
Former	27.09	25.97	28.68	
Current	17.29	10.49	26.85	
Diabetes (%)	13.74	9.38	19.87	<0.01
Periodontal treatment (%)	23.38	17.96	30.98	<0.01
Days per week using floss (%)				<0.01
0	27.53	22.47	34.64	
1–2	17.47	18.77	15.65	
3–4	14.89	15.74	13.7	
5–7	40.09	43.02	35.96	
Days per week using mouthwash (%)				<0.01
0	44.75	46.55	42.22	
1–2	10.52	12.17	8.2	
3–4	10.73	10.63	10.86	
5–7	33.99	30.65	38.68	
Calcium intake (mg)	970.96 ± 549.61	978.95 ± 540.26	959.73 ± 562.29	<0.01
Hypertension (%)	34.68	30.04	41.21	<0.01
Hyperlipidemia (%)	38.91	37.69	40.62	0.11
Congestive heart failure (%)	2.39	1.04	3.48	<0.01
Heart attack (%)	2.63	1.62	4.06	<0.01
Coronary heart disease (%)	2.39	1.39	3.8	<0.01
Angina pectoris (%)	1.71	0.87	2.89	<0.01
Stroke (%)	2.04	1.35	3.02	<0.01
Physical activity (%)				<0.01
0	21.87	19.7	24.93	
1	14.77	15.73	13.42	
2	11.64	12.55	10.37	
3	51.71	52.02	51.28	

BMI, body mass index; BMD, bone mineral density.

### Association between blood trace minerals and mean clinical attachment loss

Three weighted univariate and multivariate linear regression models were constructed: Model 1, unadjusted; Model 2, adjusted for age, sex, race/ethnicity; Model 3, adjusted for the covariables in Table 1. In the fully adjusted model, we observed a significant positive association between blood lead and cadmium and mean CAL [0.6081 (0.3211, 0.8950); 0.0266 (0.0213, 0.0318)], especially blood lead, and a negative association between blood selenium and mean CAL [−0.1056 (−0.1647, −0.0465)]. However, the associations between blood mercury, blood manganese and mean CAL were not significant [−0.0015 (−0.0035 0.0003); 0.0002 (−0.0001, 0.0006)] (Table 3). In addition, blood lead, cadmium and selenium levels were transformed into quartiles. And the mean CAL of the highest quartiles of blood lead increased by 0.15 mm compared with the lowest quartiles [0.1517 (0.0767, 0.2268)] (Table 4). The mean CAL of the highest quartiles of blood cadmium increased by 0.32 mm compared with the lowest quartiles [0.3168 (0.2286, 0.4049)] (Table 5), while for blood selenium, the mean CAL decreased by 0.15 mm [−0.1454 (−0.2118, −0.0789)] (Table 6). The overall results appeared to be roughly the same, indicating that the conclusion was relatively stable. Furthermore, all *P* for trend < 0.001 indicated that the variation trends were significant.

**TABLE 3:** Association between trace mineral concentration (μmol/L) and CAL (mm).

	Model 1 β (95% CI)	Model 2 β (95% CI)	Model 3 β (95% CI)
Blood lead	2.3426 (2.0183, 2.6669)***	1.4298 (1.1158, 1.7438)***	0.6081 (0.3211, 0.8950)***
Blood cadmium	0.0512 (0.0464, 0.0561)***	0.0535 (0.0490, 0.0580)***	0.0266 (0.0213, 0.0318)***
Blood mercury	−0.0038 (−0.0059, −0.0017)***	−0.0057 (−0.0077, −0.0038)***	−0.0015 (−0.0033, 0.0003)
Blood selenium	−0.1714 (−0.2420, −0.1008)***	−0.1948 (−0.2607, −0.1290)***	−0.1056 (−0.1647, −0.0465)***
Blood manganese	−0.0005 (−0.0009, −0.0001)**	0.0002 (−0.0002, 0.0006)	0.0002 (−0.0001, 0.0006)

Model 1, unadjusted.

Model 2, age, sex, race/ethnicity were adjusted.

Model 3, age, sex, race/ethnicity, BMI, income-poverty ratio, education, vitamin D, smoking status, diabetes, frequency per week using floss and mouthwash, periodontal treatment, hypertension, hyperlipidemia, BMD, congestive heart failure, coronary heart disease, angina pectoris, heart attack, stroke, physical activity, and calcium intake were adjusted. **P < 0.01; ***P < 0.001.

**TABLE 4:** Association between blood lead concentration (μmol/L) and CAL (mm), and stratified by sex, race/ethnicity, age and diabetes history.

	Model 1 β (95% CI)	Model 2 β (95% CI)	Model 3 β (95% CI)
Blood lead	2.3426 (2.0183, 2.6669)***	1.4298 (1.1158, 1.7438)***	0.6081 (0.3211, 0.8950)***
Lowest quartiles	Reference	Reference	Reference
2nd	0.1526 (0.0793, 0.2260)***	0.0017 (−0.0701, 0.0734)	−0.0779 (−0.1427, −0.0131)*
3rd	0.3935 (0.3178, 0.4691)***	0.1551 (0.0785, 0.2317)***	0.0158 (−0.0550, 0.0866)
4th	0.7043 (0.6283, 0.7803)***	0.3987 (0.3190, 0.4783)***	0.1517 (0.0767, 0.2268)***
*P* for trend	<0.001	<0.001	<0.001
**Stratified by sex**			
Male	1.8883 (1.4720, 2.3046)***	1.3609 (0.9599, 1.7619)***	0.4966 (0.1361, 0.8572)**
Female	2.7857 (2.1833, 3.3880)***	1.5989 (0.9964, 2.2015)***	0.6120 (0.0398, 1.1842)*
**Stratified by race/ethnicity**			
Non-Hispanic White	2.8884 (2.3317, 3.4451)***	1.8592 (1.2973, 2.4210)***	0.8259 (0.3181, 1.3337)**
Non-Hispanic Black	3.0393 (2.3438, 3.7347)***	1.8568 (1.1799, 2.5337)***	0.8896 (0.2289, 1.5503)**
Mexican American	0.7008 (0.1253, 1.2762)*	0.5346 (0.0064, 1.0627)*	0.2545 (−0.2502, 0.7592)
Other	2.4036 (1.6128, 3.1943)***	1.3249 (0.5675, 2.0823)***	0.3983 (−0.3225, 1.1191)
**Stratified by age**			
30–44 years	1.2188 (0.8313, 1.6062)***	0.9279 (0.5501, 1.3056)***	0.3966 (0.0426, 0.7506)*
45–59 years	3.0993 (2.4414, 3.7572)***	2.4614 (1.8203, 3.1025)***	1.0921 (0.5260, 1.6582)***
60–74 years	1.3067 (0.6360, 1.9773)***	0.9105 (0.2606, 1.5605)**	0.2472 (−0.3635, 0.8578)
≥75 years	2.9180 (1.0676, 4.7684)**	2.6220 (0.8521, 4.3920)**	2.0299 (0.2003, 3.8595)*
**Stratified by diabetes history**			
Diabetic	4.1482 (3.0791, 5.2173)***	3.2662 (2.2254, 4.3071)***	2.0253 (1.0549, 2.9956)***
Non-diabetic	2.1271 (1.7971, 2.4570)***	1.2641 (0.9415, 1.5866)***	0.4134 (0.1185, 0.7083)**

Model 1, unadjusted.

Model 2, age, sex, race/ethnicity were adjusted.

Model 3, age, sex, race/ethnicity, BMI, income-poverty ratio, education, vitamin D, smoking status, diabetes, frequency per week using floss and mouthwash, periodontal treatment, hypertension, hyperlipidemia, BMD, congestive heart failure, coronary heart disease, angina pectoris, heart attack, stroke, physical activity, and calcium intake were adjusted. In the subgroup analysis stratified, the model is not adjusted for the stratification variable itself. *P < 0.05; **P < 0.01; ***P < 0.001.

**TABLE 5:** Association between blood cadmium concentration (μmol/L) and CAL (mm), and stratified by sex, race/ethnicity, age, and diabetes history.

	Model 1 β (95% CI)	Model 2 β (95% CI)	Model 3 β (95% CI)
Blood cadmium	0.0512 (0.0464, 0.0561)***	0.0535 (0.0490, 0.0580)***	0.0266 (0.0213, 0.0318)***
Lowest quartiles	Reference	Reference	Reference
2nd	0.0420 (−0.0295, 0.1136)	0.0211 (−0.0461, 0.0882)	−0.0046 (−0.0676, 0.0584)
3rd	0.1890 (0.1149, 0.2632)***	0.1367 (0.0650, 0.2084)***	0.0255 (−0.0437, 0.0946)
4th	0.7677 (0.6918, 0.8436)***	0.7468 (0.6751, 0.8184)***	0.3168 (0.2286, 0.4049)***
*P* for trend	<0.001	<0.001	<0.001
**Stratified by sex**			
Male	0.0796 (0.0713, 0.0879)***	0.0769 (0.0690, 0.0847)***	0.0418 (0.0324, 0.0511)***
Female	0.0344 (0.0292, 0.0396)***	0.0356 (0.0307, 0.0405)***	0.0181 (0.0123, 0.0239)***
**Stratified by race/ethnicity**			
Non-Hispanic White	0.0515 (0.0450, 0.0580)***	0.0546 (0.0485, 0.0606)***	0.0258 (0.0186, 0.0329)***
Non-Hispanic Black	0.0493 (0.0349, 0.0638)***	0.0493 (0.0361, 0.0625)***	0.0201 (0.0032, 0.0370)*
Mexican American	0.0745 (0.0446, 0.1045)***	0.0733 (0.0456, 0.1011)***	0.0570 (0.0243, 0.0898)***
Other	0.0426 (0.0314, 0.0537)***	0.0391 (0.0287, 0.0495)***	0.0230 (0.0112, 0.0348)***
**Stratified by age**			
30–44 years	0.0291 (0.0234, 0.0349)***	0.0311 (0.0255, 0.0366)***	0.0130 (0.0064, 0.0196)***
45–59 years	0.0539 (0.0462, 0.0617)***	0.0584 (0.0511, 0.0657)***	0.0294 (0.0210, 0.0377)***
60–74 years	0.1030 (0.0881, 0.1179)***	0.0989 (0.0847, 0.1132)***	0.0558 (0.0379, 0.0738)***
≥75 years	0.0777 (0.0342, 0.1212)***	0.0820 (0.0413, 0.1227)***	0.0350 (−0.0088, 0.0789)
**Stratified by diabetes history**			
Diabetic	0.0771 (0.0603, 0.0940)***	0.0761 (0.0601, 0.0922)***	0.0538 (0.0359, 0.0716)***
Non-diabetic	0.0495 (0.0446, 0.0544)***	0.0514 (0.0469, 0.0559)***	0.0235 (0.0181, 0.0289)***

Model 1, unadjusted.

Model 2, age, sex, race/ethnicity were adjusted.

Model 3, age, sex, race/ethnicity, BMI, income-poverty ratio, education, vitamin D, smoking status, diabetes, frequency per week using floss and mouthwash, periodontal treatment, hypertension, hyperlipidemia, BMD, congestive heart failure, coronary heart disease, angina pectoris, heart attack, stroke, physical activity, and calcium intake were adjusted. In the subgroup analysis stratified, the model is not adjusted for the stratification variable itself. *P < 0.05; ***P < 0.001.

**TABLE 6:** Association between blood selenium concentration (μmol/L) and CAL (mm), and stratified by sex, race/ethnicity, age, and diabetes history.

	Model 1 β (95% CI)	Model 2 β (95% CI)	Model 3 β (95% CI)
Blood selenium	−0.1714 (−0.2420, −0.1008)***	−0.1948 (−0.2607, −0.1290)***	−0.1056 (−0.1647, −0.0465)***
Lowest quartiles	Reference	Reference	Reference
2nd	−0.1900 (−0.2700, −0.1100)***	−0.1921 (−0.2665, −0.1177)***	−0.1057 (−0.1720, −0.0395)**
3rd	−0.1870 (−0.2653, −0.1086)***	−0.2014 (−0.2744, −0.1284)***	−0.0869 (−0.1524, −0.0215)**
4th	−0.2319 (−0.3113, −0.1525)***	−0.2597 (−0.3339, −0.1856)***	−0.1454 (−0.2118, −0.0789)***
*P* for trend	<0.001	<0.001	<0.001
Stratified by sex			
Male	−0.3919 (−0.5153, −0.2686)***	−0.3659 (−0.4825, −0.2493)***	−0.1974 (−0.3007, −0.0942)***
Female	−0.0787 (−0.1534, −0.0040)*	−0.0686 (−0.1392, 0.0020)	−0.0346 (−0.0995, 0.0303)
Stratified by race/ethnicity			
Non−Hispanic White	−0.1427 (−0.2386, −0.0468)**	−0.1794 (−0.2705, −0.0882)***	−0.1195 (−0.1985, −0.0405)**
Non-Hispanic Black	−0.1638 (−0.4045, 0.0769)	−0.2661 (−0.4877, −0.0446)*	−0.0858 (−0.2944, 0.1227)
Mexican American	0.0040 (−0.3188, 0.3268)	−0.1024 (−0.3991, 0.1943)	0.1235 (−0.1672, 0.4143)
Other	−0.1990 (−0.3484, −0.0496)**	−0.2783 (−0.4173, −0.1393)***	−0.1629 (−0.2941, −0.0317)*
Stratified by age			
30–44 years	−0.1716 (−0.2747, −0.0684)**	−0.2035 (−0.3034, −0.1037)***	−0.1581 (−0.2516, −0.0646)***
45–59 years	−0.3407 (−0.4976, −0.1838)***	−0.3245 (−0.4743, −0.1746)***	−0.1233 (−0.2525, 0.0060)
60–74 years	−0.0989 (−0.2097, 0.0119)	−0.1050 (−0.2105, 0.0004)	−0.0662 (−0.1582, 0.0259)
≥75 years	−0.1482 (−0.4747, 0.1783)	−0.1818 (−0.4893, 0.1257)	−0.1573 (−0.4602, 0.1456)
Stratified by diabetes history			
Diabetic	−0.3435 (−0.5703, −0.1166)**	−0.3064 (−0.5248, −0.0881)**	−0.0854 (−0.2871, 0.1164)
Non-diabetic	−0.1557 (−0.2278, −0.0835)***	−0.1828 (−0.2504, −0.1153)***	−0.1130 (−0.1733, −0.0527)***

Model 1, unadjusted.

Model 2, age, sex, race/ethnicity were adjusted.

Model 3, age, sex, race/ethnicity, BMI, income-poverty ratio, education, vitamin D, smoking status, diabetes, frequency per week using floss and mouthwash, periodontal treatment, hypertension, hyperlipidemia, BMD, congestive heart failure, coronary heart disease, angina pectoris, heart attack, stroke, physical activity, and calcium intake were adjusted. In the subgroup analysis stratified, the model is not adjusted for the stratification variable itself. *P < 0.05; **P < 0.01; ***P < 0.001.

### Subgroup analyses of blood cadmium, blood lead, blood selenium, and mean clinical attachment loss

Further subgroup analysis of blood lead showed that the β value in females was slightly higher than males (Table 4). The association was significant in White and Black people [0.8259 (0.3181, 1.3337); 0.8896 (0.2289, 1.5503)], but not in other race/ethnicity. The strongest association was observed in the elderly with ≥5 years [2.0299 (0.2003, 3.8595)]. The β value in the diabetic group was almost five times higher than that in the non-diabetic group [2.0253 (1.0549, 2.9956); 0.4134 (0.1185, 0.7083)]. All subgroups were statistically different in blood cadmium (Table 5). The β value of males was significantly higher than that of females [0.0418 (0.0324, 0.0511); 0.0181 (0.0123, 0.0239)], and the association was strongest in Mexican-Americans [0.0570 (0.0243, 0.0898)]. Among the different age groups, the β value of the 60–74 years old was the highest [0.0558 (0.0379, 0.0738)]. The β value of the diabetic group was almost twice that of the non-diabetic group [0.0538 (0.0359, 0.0716); 0.0235 (0.0181, 0.0289)]. For blood selenium (Table 6), we observed a negative association between blood selenium and mean CAL in males [−0.1974 (−0.3007, −0.0942)], while it was not significant in females [−0.0346 (−0.0995, 0.0303)]. For people aged 30–44 [−0.1581 (−0.2516, −0.0646)] and without diabetes [−0.1130 (−0.1733, −0.0527)], the negative association was statistically significant.

### Curve fit analysis

**FIGURE 2:**
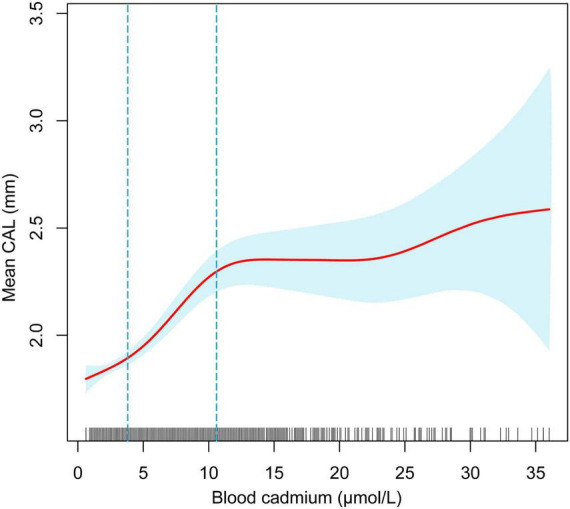
The non-linear relationship between blood cadmium and mean CAL. The blue area represents the 95% confidence interval. The red line represents point estimates. Age, sex, race/ethnicity, BMI, income-poverty ratio, education, vitamin D, smoking status, diabetes, frequency per week using floss and mouthwash, periodontal treatment, hypertension, hyperlipidemia, BMD, congestive heart failure, coronary heart disease, angina pectoris, heart attack, stroke, physical activity, and calcium intake were adjusted.

**FIGURE 3:**
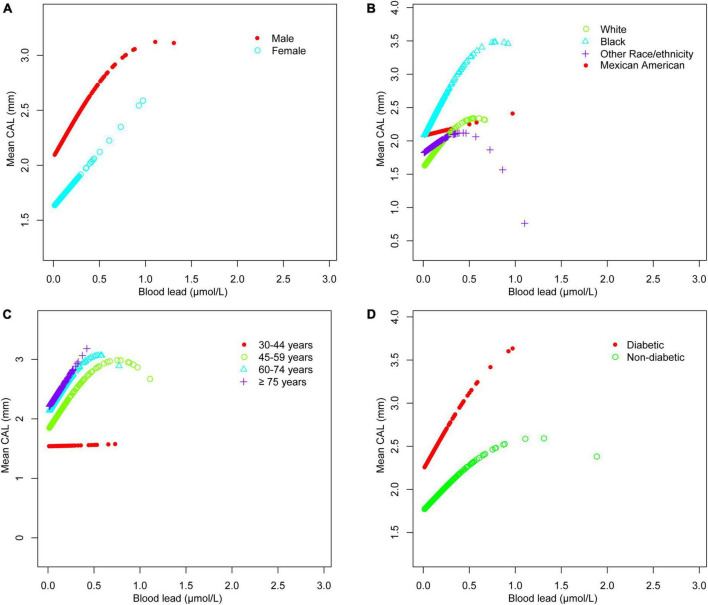
Blood lead and mean CAL dose-response relationship. **(A)** Stratified by sex. **(B)** Stratified by race/ethnicity. **(C)** Stratified by age. **(D)** Stratified by diabetes history. Age, sex, race/ethnicity, BMI, income-poverty ratio, education, vitamin D, smoking status, diabetes, frequency per week using floss and mouthwash, periodontal treatment, hypertension, hyperlipidemia, BMD, congestive heart failure, coronary heart disease, angina pectoris, heart attack, stroke, physical activity, and calcium intake were adjusted. In the subgroup analysis stratified, the stratification variable itself was not adjusted.

**FIGURE 4:**
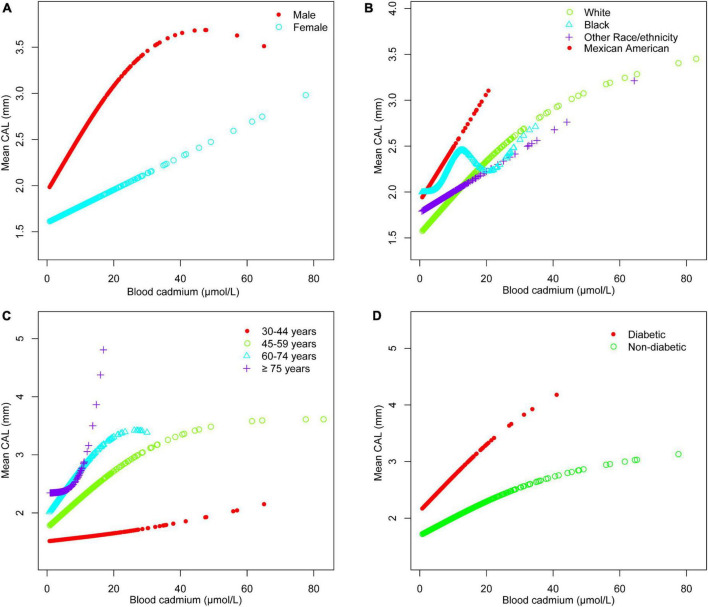
Blood cadmium and mean CAL dose-response relationship. **(A)** Stratified by sex. **(B)** Stratified by race/ethnicity. **(C)** Stratified by age. **(D)** Stratified by diabetes history. Age, sex, race/ethnicity, BMI, income-poverty ratio, education, vitamin D, smoking status, diabetes, frequency per week using floss and mouthwash, periodontal treatment, hypertension, hyperlipidemia, BMD, congestive heart failure, coronary heart disease, angina pectoris, heart attack, stroke, physical activity, and calcium intake were adjusted. In the subgroup analysis stratified, the stratification variable itself was not adjusted.

The adjusted smoothing curve showed a non-linear relationship between blood cadmium and mean CAL (Figure 2). Based on the piecewise linear regression model, the turning points were calculated to be 4.03 and 10.32 μmol/L, respectively (Table 7). When the blood cadmium level was <4.03 μmol/L, the β value was very small and the association was not significant [0.0152 (−0.0143, 0.0447)], until beyond 4.03 μmol/L, a threshold effect occurred, which was positively associated with mean CAL [0.0889 (0.0550, 0.1228)]. After the blood cadmium level reached 10.32 μmol/L, the β value decreased greatly and became saturated [0.0178 (0.0073, 0.0284)]. Stratified by age, sex, race/ethnicity, and diabetes history, and further using smooth curve fitting analysis to determine the non-linear relationship between blood lead, blood cadmium, blood selenium and mean CAL, we discovered that there was an obvious turning point in blood lead among people aged 45–59 and in blood cadmium among Black people (Table 7). The mean CAL increased with the increase in blood lead among people aged 45–59 [6.6634 (3.4448, 9.8820)], until the turning point (0.065 μmol/L) (Table 7; Figure 3). Similarly, the relationship between blood cadmium and mean CAL in Black people followed an inverted U-shaped curve with a turning point of 12.99 μmol/L (Table 7; Figure 4). There was an almost linear relationship between the blood selenium subgroups and mean CAL (Supplementary Figure 1).

**TABLE 7:** Threshold effect and saturation effect analysis of blood cadmium and blood lead on mean CAL.

Mean CAL (mm)	Adjusted ß (95% CI), *P*-value
Blood cadmium < 4.03 (μmol/L)	0.0152 (−0.0143, 0.0447), 0.3115
4.03 (μmol/L) < Blood cadmium < 10.32 (μmol/L)	0.0889 (0.0550, 0.1228), <0.0001
Blood cadmium > 10.32 (μmol/L)	0.0178 (0.0073, 0.0284), 0.001
**Non-Hispanic Black**	
Blood cadmium < 12.99 (μmol/L)	0.0508 (0.0222, 0.0794), 0.0005
Blood cadmium > 12.99 (μmol/L)	−0.0130 (−0.0431, 0.0171), 0.3980
**45–59 years**	
Blood lead < 0.065 (μmol/L)	6.6634 (3.4448, 9.8820), <0.0001
Blood lead > 0.065 (μmol/L)	0.6459 (0.0354, 1.2564), 0.0383

Age, sex, race/ethnicity, BMI, income-poverty ratio, education, vitamin D, smoking status, diabetes, frequency per week using floss and mouthwash, periodontal treatment, hypertension, hyperlipidemia, BMD, congestive heart failure, coronary heart disease, angina pectoris, heart attack, stroke, physical activity, and calcium intake were adjusted. In the subgroup analysis stratified, the model is not adjusted for the stratification variable itself.

### Association between blood trace minerals and periodontal status

The relationship between blood trace mineral levels and periodontal status was determined using multinomial logistic regression (Table 8). Blood lead and cadmium levels showed significant differences in different periodontal statuses. Compared with people without periodontitis, for every unit increase in blood lead, the risk of moderate periodontitis increased by 3.14 times [4.143 (1.233, 13.921)], and the risk of severe periodontitis increased by 27.15 times [28.145 (7.112, 111.376)]. For every unit increase in blood cadmium, the incidence of severe periodontitis was 1.06 times higher than that of non-periodontitis [1.058 (1.036, 1.08)].

**TABLE 8:** Association between trace mineral concentration (μmol/L) and periodontal status (mm).

	No periodontitis	Mild periodontitis	Moderate periodontitis	Severe periodontitis
Blood lead	1.0	1.077 (0.024, 49.176)	4.143 (1.233, 13.921)	28.145 (7.112, 111.376)
Blood cadmium	1.0	0.924 (0.853, 1.002)	1.013 (0.995, 1.031)	1.058 (1.036, 1.08)
Blood mercury	1.0	0.987 (0.967, 1.007)	1.0 (0.995, 1.005)	0.993 (0.984, 1.002)
Blood selenium	1.0	1.29 (0.812, 2.049)	0.908 (0.748, 1.101)	0.782 (0.570, 1.072)
Blood manganese	1.0	0.999 (0.996, 1.002)	1.0 (0.999,1.001)	1.0 (0.98, 1.001)

No periodontitis was considered as reference group. Age, sex, race/ethnicity, BMI, income-poverty ratio, education, vitamin D, smoking status, diabetes, frequency per week using floss and mouthwash, periodontal treatment, hypertension, hyperlipidemia, BMD, congestive heart failure, coronary heart disease, angina pectoris, heart attack, stroke, physical activity, and calcium intake were adjusted.

## Discussion

Heavy metals are known to have long-term adverse health effects, but exposure continues, especially in less developed countries and communities (25). Lead exposure mainly comes from informal battery recycling and manufacturinge, electronic waste, metal mining, and food adulteration, especially in spices. A study compiled background values of blood lead levels of children in 34 countries to estimate a background distribution of 1,300 million of them. It was estimated that 48.5% of the children (632 million) had blood lead levels exceeding the 5 μg/dL reference value of CDC (26). Agricultural and industrial sources contribute to the presence of cadmium in the environment as a pollutant. Cadmium accumulates in plants and animals, with a long half-life of approximately 25–30 years. Cigarette smoke is the primary source of exposure to cadmium in smokers because tobacco leaves accumulate high levels of cadmium in the soil (27). In non-smokers, cadmium is most commonly ingested through food and water contaminated with cadmium (28). Selenium is an essential trace element. Currently, the recommended daily selenium allowance is 55 μg/day (29). Most people are exposed to selenium through food, water and air. Selenium enters the food chain through plants, and low dietary selenium intake in humans is related to crops grown in selenium-deficient soils. Therefore, animal food can be used as an alternative source because animals can accumulate selenium from feed supplemented with selenium or pastures with selenium fertilizers (30). The general population is mainly exposed to mercury through food, fish, and dental amalgams. Manganese is primarily consumed in the diet; however, occupational exposure to manganese may also occur.

By analyzing a nationally representative sample of adults in the United States, this study investigated the relationship between blood trace mineral concentrations and periodontitis (mean CAL and periodontal status). Linear regression analyses showed a positive association between blood lead, blood cadmium, and mean CAL, and a negative association between blood selenium, blood mercury, blood manganese, and mean CAL. Even after adjusting for all other factors to minimize potential confounding, blood lead, blood cadmium, and blood selenium levels were still significantly associated with mean CAL, but there was no significant association between blood mercury, blood manganese, and mean CAL (Table 3). The results of regression analyses of quartiles of blood lead, blood cadmium, and blood selenium, as well as the results of trend tests (*P* for trend < 0.001) both confirmed their association with mean CAL (Tables 4–6). There was a significant positive association between blood lead and cadmium levels and periodontitis severity in the fully adjusted model by multinomial logistic regression. Therefore, the next research focus was on the association between blood lead, cadmium, selenium, and periodontitis.

Periodontitis leads to the loss of alveolar bone and support of the periodontal ligament (31). Clinical attachment loss is the direct result of periodontal supporting tissue destruction, a vital sign to distinguish gingivitis from periodontitis, and an essential index for the clinical diagnosis and grading of periodontitis. Periodontal pocket depth may cause errors owing to gingival inflammation, resulting in false-positive results. In contrast, clinical attachment loss can reflect the degree of periodontal tissue destruction more objectively and genuinely.

Periodontitis is a complex infectious disease that is affected by many factors such as diabetes history, smoking status, age, stroke and inadequate oral hygiene (32, 33). The imbalance of trace minerals may be part of the pathogenic pathway and play a unique role in the complex etiology and mechanism networks. Various subgroup analyses were performed after controlling for confounding variables. Surprisingly, the threshold effect between blood cadmium and mean CAL was determined by curve fitting and piecewise linear regression. When blood cadmium reached 4.03 μmol/L, the mean CAL began to increase with the increase in blood cadmium concentration. This suggests that people should maintain blood cadmium below 4.03 μmol/L as much as possible, which is beneficial to periodontal health. Subgroup analyses showed that the β values of blood cadmium and blood selenium in males were significantly higher than females. There was a significant positive association between blood lead and mean CAL in the elderly, with a β value of 2.03, indicating that the elderly should pay special attention to the control of blood lead levels, which is helpful in maintaining periodontal health. For White and Black people, there was a significant positive association between blood lead and mean CAL. Similarly, the association between blood cadmium levels and mean CAL was stronger in Mexican Americans than in other populations. Interestingly, the β values of blood lead, and blood cadmium in participants with diabetes were much higher than those in participants without diabetes. In contrast, the association between blood selenium and mean CAL was not affected in those people, indicating that patients with diabetes had a higher risk of periodontitis, even from the perspective of trace minerals. There was a non-linear relationship between blood cadmium and mean CAL. Among the Black population, the relationship between blood cadmium levels and mean CAL followed an inverted U-shaped curve. There was a saturation effect in the study of blood lead in people aged 45–59 years old.

Lead exposure can cause dysbacteriosis in dental plaques (34). Among children exposed to severe lead pollution, the prevalence rate of *A. actinomycetemcomitans* infection is as high as 17%, which is the primary pathogen associated with juvenile periodontitis (11, 35). Lead can also damage various biochemical processes, including inhibition of calcium and reaction with proteins. Even at low blood lead levels, lead may inhibit the activity of various enzymes by competing with the necessary cations of binding sites, causing structural changes, such as glucose 6-phosphate dehydrogenase, catalase superoxide dismutase, glutathione peroxidase, and antioxidants such as glutathione (19). Additionally, lead can induce the production of ROS and oxidative stress, which will further lead to the deterioration of dental health. Nattaporn et al. (11) investigated the periodontal status of children in a lead-polluted environment, finding that the high-lead group showed more deep pockets at tooth 46 and tooth 16 than the low-lead group.

The level of cadmium in human cadaver mandibles was measured, and it was found that there may be an association between the level of cadmium in basal bone and the presence of periodontitis. In addition, alveolar bone in the mandible accumulated higher amounts of cadmium than the basal bone, which might impact the progression of skeletal changes associated with periodontitis (36). Cadmium induces oxidative stress and ROS production, causing the oxidation and damage of biologically important macromolecules, such as lipids, proteins, DNA, and cellular membrane phospholipids. Cadmium may interfere with the activity of antioxidant enzymes, such as manganese superoxide dismutase, catalase, and copper-zinc superoxide dismutase which is a zinc-oncentrating protein that can be used as a free-radical scavenger (37), further aggravating the damage of oxidative stress. In addition, cadmium can destroy signaling cascades and cause a variety of toxic effects, mainly because of its physicochemical similarity to the calcium ion. It can destroy calcium-mediated signaling pathways, which may be achieved by significantly changing the activation of calmodulin and calmodulin-dependent protein kinase II in cell death pathways, such as apoptosis, necrosis or autophagy (38).

Selenium may be essential to periodontal tissue. It had been found that, in *in vitro* and animal experiments, adding selenium to α-Tocopherol could accelerate cell proliferation and wound healing. his might be due to selenium promoting the synthesis of basic fibroblast growth factor and type I collagen by gingival and periodontal ligament fibroblasts (39). Selenium can prevent the aggravation of the immune response in chronic inflammation. At the cellular level, selenium may affect the function of various leukocyte effectors, including adhesion, migration, phagocytosis and secretion of cytokines (40). The glutathione peroxidase system is the most widely studied, which uses the selenium of its active site to detoxify ROS, such as hydrogen peroxide and phospholipid hydrogen peroxide. Similarly, selenium regulates the activity of transcription factors (nuclear factor-kappa B and activator protein-1) and the expression of related genes (41). Selenium even reduces levels of tumor necrosis factor-α and cyclooxygenase-2 produced by macrophages in response to endotoxins and down-regulates the expression of adhesion molecules. In addition, it also contributes to the metabolism of arachidonic acid and eicosanes (42).

In the present study, we analyzed a representative sample of multiracial populations for better generalizability to the United States population. In addition, participants were evaluated and operated by trained staff, and interviews were conducted following standardized procedures and strict quality control to obtain examination data and laboratory data, which improved the accuracy and validity of the data. In the data analysis, we adjusted for a considerable number of potential confounding variables. Such a large sample size enabled us to carry out further subgroup and piecewise model analyses. This is the main advantage of this study. In addition, the potential association between trace elements and periodontitis found in this study may help scholars to carry out more randomized controlled trials in related aspects because, at present, only a few clinical trials have investigated their relationship. However, the present study had several limitations. First, it had a cross-sectional design, and the direction of the association between trace minerals and periodontitis was difficult to explain as causality. We could not infer long-term trends of periodontal status because all measurements of an object were taken at the same time. Second, although we adjusted for more than a dozen major covariates in the analysis, we might have omitted some possible residual confounders that were not taken into account when the model was designed or for which there was not enough information in the database to allow proper adjustments. For example, NHANES 2011–2012 and 2013–2014 did not collect information such as plaque index, dental calculus index, bad oral habits, probing bleeding, and parents’ periodontal history. Third, the data source of NHANES database is the United States population. Therefore, the findings should be extrapolated cautiously to other populations in different countries. The mechanism underlying the relationship between trace minerals and periodontitis requires further study. Nevertheless, the role of trace minerals in periodontitis should be emphasized because it is relatively easy to control trace minerals in the general population. Individuals with periodontitis require special attention to diet and environment to improve their trace mineral levels, which may help alleviate periodontitis.

## Conclusion

Blood lead and cadmium levels were positively associated with mean CAL, and blood selenium was negatively associated with mean CAL; however, blood mercury, blood manganese, and mean CAL were not significantly associated. The association between trace minerals and mean CAL was more significant in males, the elderly, and patients with diabetes. There was a threshold effect between blood cadmium levels and mean CAL. Among the Black population, the relationship between blood cadmium levels and mean CAL followed an inverted U-shaped curve. There was a saturation effect in the study of blood lead in people aged 45–59 years old. Blood selenium, lead, and cadmium levels were significantly associated with periodontitis. Due to the limitations of this study, further research is needed, including mechanistic experiments and large-sample multicenter prospective studies, to explore the pathogenesis behind this special relationship.

## Data availability statement

Publicly available datasets were analyzed in this study. This data can be found here: www.cdc.gov/nchs/nhanes/.

## Author contributions

HH, JYa, and NY contributed to the conception and design of the study. HH, LY, LT, JYu, and YG contributed to drafting the article. ZL contributed to revising the article critically. All the authors have read and approved the manuscript.

## Footnotes

1
http://www.health.gov/paguidelines/

2
http://www.R-project.org

3
http://www.empowerstates.com
