# Supplementary material for: Association between levels of blood trace minerals and periodontitis among United States adults
Source: Front Nutr. 2022 Sep 7;9:999836. doi: 10.3389/fnut.2022.999836 (PMC9490086; doi:10.3389/fnut.2022.999836)
Supplement: Supplementary file 1 [file Table_1.DOCX]

Supplementary Material

# Supplementary Figures


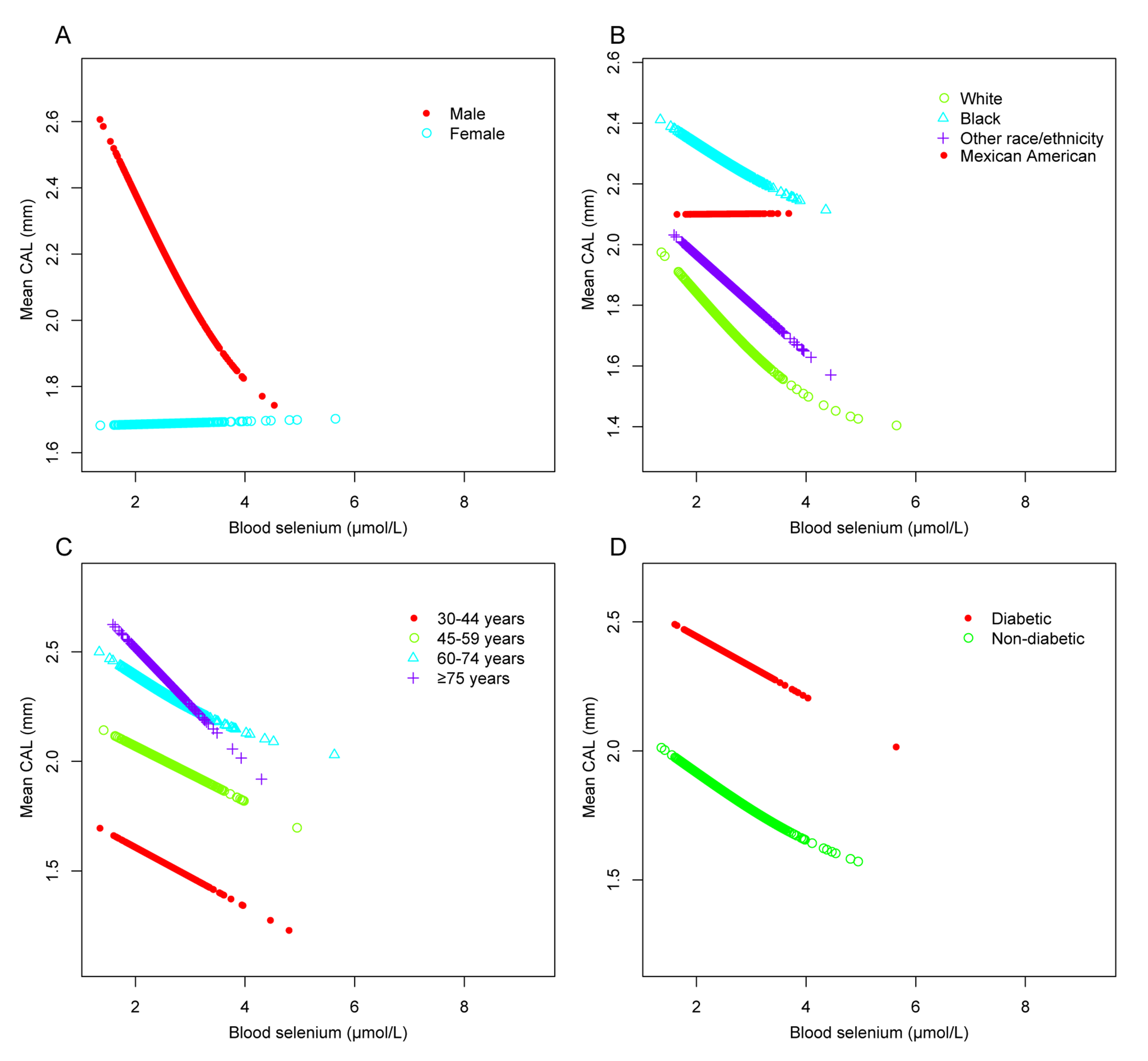


**Supplementary Figure 1.** Blood selenium and mean CAL dose–response relationship. (A) Stratified by Sex. (B) Stratified by race/ethnicity. (C) Stratified by age. (D) Stratified by diabetes history. Age, sex, race/ethnicity, BMI, income-poverty ratio, education, vitamin D, smoking status, diabetes, frequency per week using floss and mouthwash, periodontal treatment, hypertension, hyperlipidemia, BMD, congestive heart failure, coronary heart disease, angina pectoris, heart attack, stroke, physical activity and calcium intake were adjusted. In the subgroup analysis stratified, the stratification variable itself was not adjusted.
